# Increases of Quadriceps Inter-Muscular Cross-Correlation and Coherence during Exhausting Stepping Exercise

**DOI:** 10.3390/s121216353

**Published:** 2012-11-26

**Authors:** Ya-Ju Chang, Ching-Chieh Chou, Hsiao-Lung Chan, Miao-Ju Hsu, Ming-Yuh Yeh, Chia-Ying Fang, Yu-Fen Chuang, Shun-Hwa Wei, Hen-Yu Lien

**Affiliations:** 1 Department of Physical Therapy and Graduate Institute of Rehabilitation Science, Chang Gung University, 259, Wen-Hwa 1st Rd, Kweishan, Taoyuan 333, Taiwan; E-Mails: yjchang@mail.cgu.edu.tw (Y.-J.C.); u8606034@yahoo.com.tw (C.-Y.F.); chuang@mail.cgu.edu.tw (Y.-F.C.); hyl@mail.cgu.edu.tw (H.-Y.L.); 2 Department of Electrical Engineering, Chang Gung University, 259, Wen-Hwa 1st Rd, Kweishan, Taoyuan 333, Taiwan; E-Mail: d9621003@stmail.cgu.edu.tw; 3 Department of Physical Therapy, College of Health Science, Kaohsiung Medical University, 100,Shih-Chuan 1st Road, Kaohsiung 80708, Taiwan; E-Mail: mjhsu@kmu.edu.tw; 4 Department of Physical Medicine and Rehabilitation, Kaohsiung Medical University Hospital, 100, Tzyou 1st Road, Kaohsiung 807, Taiwan; 5 Department of Rehabilitation Technology, Tzu Hui Institute of Technology, 367, Sanmin Road, Nanjhou Hsian, Pingtung 926, Taiwan; 6 Department of Physical Therapy and Assistive Technology, National Yang Ming University, 155, Sec.2, Linong Street, Taipei 112, Taiwan; E-Mail: shunhwa@ym.edu.tw

**Keywords:** intermuscular, cross-correlation, coherence, muscle fatigue and stepping

## Abstract

The aim of this study was to examine the change of the intermuscular cross-correlation and coherence of the *rectus femoris* (RF), *vastus medialis* (VM) and *vastus lateralis* (VL) during exhausting stepping exercise. Eleven healthy adults repeated the stepping exercise up to their individual endurance limits (RPE score reached 20), and the cross-correlation and coherence were assessed by surface electromyography (EMG) recordings. The coefficient and time lag of cross-correlation and the coherence areas in the alpha (8–12 Hz), beta (15–30 Hz), gamma (30–60 Hz) and high-gamma (60–150 Hz) bands among the three muscle pairs (RF-VM, RF-VL and VM-VL) were calculated. As muscle fatigue, RF-VM and VM-VL showed increases of coefficients and the shortening of time lags. RF-VM and RF-VL showed increases of beta-band coherence in the ascent and descent phases, respectively. The increased intermuscular cross-correlation and beta-band coherence may be a compensatory strategy for maintaining the coordination of knee synergistic muscles during fatigue due to the fatigue-related disturbance of the corticospinal transmission. Therefore, the intermuscular cross-correlation and beta-band coherence may be a potential index for assessing muscle fatigue and monitoring the central control of motor function during dynamic fatiguing exercise.

## 1. Introduction

Stepping exercises are a common form of training that involves repetitive shortening and lengthening contractions of the quadriceps muscles. Considering the repetitive movement features, the increasing possibility of muscle injury in the quadriceps may relate to fatigue [1,2]. To avoid causing excessive fatigue and to maintain the maximal training effect, the dose setting for the stepping exercise should be carefully chosen. Therefore, knowing the neuromuscular control mechanism and quantifying the fatigue response during stepping exercises are important references to prescribe an appropriate exercise intensity.

Fatigue is defined as a decline in the capacity of a muscle to generate maximal force, and several studies have estimated the contributions of central and peripheral factors to fatigue during maximal voluntary efforts [3,4]. Related to the peripheral factors, our previous work found a decline of median frequency of the electromyography (EMG) power spectrum at the end of exhausting stepping exercises [5]. The shifting of median frequency to a lower power spectrum is a well-known index of muscle fatigue, and its association with several possible mechanisms has been proposed, including the reduction of the motor-unit action potential propagating velocity [6], the change in the shape of the intracellular action potential [7], the change of motor-unit discharge patterns [8], and neuromuscular transmission failure as well as excitation-contraction coupling failure [9,10].

Related to the central factors, one of these factors is the impairment of transmission from cerebral cortices to the motor neuron pools of spinal cord [11,12]. The failure of cortex controlling spinal motor neuron pools has been studied and quantitated by corticomuscular coherence. Coherence analysis is a powerful tool to assess the synchrony of oscillatory activities, so when the rhythmic signals are transmitted from cortices down to muscles, the use of coherence analysis can reflect the functional relationship between cerebral cortices and motor control. Electroencephalography (EEG)- and magnetoencephalography (MEG)-EMG provide robust evidence for the corticomuscular synchrony [13–16]. Corticomuscular coherence has been used to estimate the intact supraspinal structures [14,15] and descending tracts, such as corticospinal tract, because the disappearance of coherence amplitude in the beta band (15–30 Hz) was found in patients with stroke and spinal cord injuries [17,18]. Additionally, corticomuscular coherence is also task-dependent in given frequency bands. At the 8–12 Hz (alpha band), it was associated with isometric contraction [14]. The 15–30 Hz (beta band) and 30–60 Hz (gamma band) were predominantly driven by the primary motor and sensory cortex [14,19], and for their functional role, these two frequency bands were associated with isometric submaximal voluntary contractions (sub-MVCs), so coherence analysis can help assess the integrity of corticomuscular pathways and establish the functional roles of the oscillatory activities from cerebral cortices. As an alternative way to assess the integrity of corticomuscular signal transmission, intermuscular coherence has been found, especially in the beta band, to be sensitive to upper motor neuron function [20], and has been used to estimate neural synchronization [8,21–23]. The effect of neural synchronization on muscle fatigue has recently been noticed. Through surface EMG recordings, a significant increase in the coefficient of cross-correlation within the vastus lateralis and in coherence area among hand and leg muscles were observed during fatiguing sustained sub-MVC [23–25]. Increased intermuscular coherence in the 15–35 Hz and 35–60 Hz frequency bands was found during sustained fatiguing contraction of hand muscles at 30% MVC [25]. Boonstra *et al.* examined the effects of fatigue on intermuscular coherence of quadriceps muscles during sustained sub-maximal contractions, and found that the coherence increase at 6–11 Hz was fatigue-related [23]. These results show that intermuscular cross-correlation and coherence are sensitive to fatigue-related changes during isometric sub-MVC, and could be a potential novel index to detect muscle fatigue.

Although fatigue-related muscle injuries are frequently found during dynamic contractions, such as quadriceps injury during dynamic and repetitive stretch-shortening contractions, there is a lack of evidence related to the change of intermuscular cross-correlation and coherence during the fatigue of stretch-shortening contractions. Therefore, the aim of this study was to investigate the fatigue-related changes of the intermuscular cross-correlation and coherence between surface EMG signals of the *rectus femoris* (RF), *vastus medialis* (VM) and *vastus lateralis* (VL) during exhausting stepping exercise. We hypothesized that the coefficient of cross-correlation and coherence area in the beta band would increase with the development of muscle fatigue during stepping exercise. The results of this study could help developing a non-invasive EMG index to monitor the controlling strategy of synergistic knee muscles during dynamic fatigue.

## 2. Methods

### 2.1. Participants

Eleven healthy adults (averaged age: 23.6 ± 3.2 y) including six males and five females participated in this study (Table 1). All of them had no any previous history of neural and musculoskeletal disease. The subjects understood the experiment procedure before they gave written consent forms approved by Chang Gung Memorial Hospital Human Ethics Committee.

### 2.2. Data Recording

The EMG signals from RF, VM and VL were recorded by three bipolar surface electrodes (B&L Engineering, Santa Ana, CA, USA) with a fixed inter-electrode distance of 2 cm. Following skin abrasion, electrodes were placed on the muscle bellies and secured with adhesive tapes. The RF electrode was placed at about 6 cm proximal to the superior border of the patella parallel with the long axis of the muscle. The VM and VL electrodes were located on the half-way of the muscle bellies [26]. The reference electrode was placed over the surface of the tibial bone. EMG signals were pre-amplified by a factor of 350 and amplified at the mainframe amplifier, which is the one with an input impedance >10 MΩ, a CMRR of 100 dB at 60 Hz, and a gain range of 0.5–100,000 times (Gould Inc., Valley View, OH, USA). The gain was set at 1,750 (350 × 5) times and the filter were set from DC to 1 kHz for this study. Signals were digitized by a 12-bit resolution A/D converter (Metrabyte DAS 1600, Keithley Instruments, Inc; Cleveland, OH, USA) at a sampling rate of 5,000 Hz. An electrogoniometer (Biometrics Ltd, Newport, UK) was placed on the medial side of a right knee joint for measuring the changes of the knee joint angle during stepping exercise.

### 2.3. Experimental Procedures

Isometric maximum voluntary contraction (MVC), a non-fatiguing task, was performed before and 1 min, 10 min, 20 min, and 30 min after the stepping exercise. In each test, subjects sat on chairs and exerted 5-sec isometric MVCs twice at 60 degree of knee flexion. The force signal was measured by a force transducer (AWU-250, Genisco Technology, Compton, CA, USA) electronically coupled to a transducer amplifier (Gould Inc.) with a gain range from 10 to 500 and a frequency response from dc to 1,000 Hz. [27], monitored online by an oscilloscope and digitized using an analog-to-digital converter with a 12-bit resolution (Metrabyte DAS 1600) at 5,000 Hz. The force measured before the stepping exercise was as the reference value (pre-fatigue condition) and after exercise was used to trace the force recovery (post-fatigue condition).

Stepping exercise, a fatiguing task, was used to assess the fatiguing effects of dynamic and repetitive contractions of RF, VM and VL. Subjects stepped up and down a 23-cm-high stair with their right bare feet at a constant speed, which was determined by a metronome, toward their individual endurance limit. They wore ankle-foot orthoses on their left feet to avoid ankle plantar flexion to ensure the full exertion of the right limb muscles. During the stepping exercise, subjects finished one single trial by performing four step cycles per 10 s and then reported the RPE score. This routine exercise was continued until they felt exhausted (RPE score reached 20), and then ten more step cycles were needed. The EMG signals and the range of motion of right knee joints were simultaneously recorded during the stepping exercise.

### 2.4. Data Reduction

The force (4th order Butterworth low-pass filter: 20 Hz) and EMG signals (4th order Butterworth band-pass filter: 5–300 Hz and band-stop filter: 60 Hz) were filtered off-line. For normalizing the changes of force and root-mean-square values of EMG signals (rEMG) before and 1 min, 10 min, 20 min, and 30 min after the stepping exercise, the values obtained before the stepping exercise were considered the reference values, and the values after the stepping exercise were normalized with respect to the reference values for each subject. During the stepping exercise, the force and EMG data acquired in the first trial were compared with that in the final trial for fatigue purpose. Within one step cycle, the EMG signals were divided into the step ascent and descent phases according to the programming judgment on the range of motion of the knee joint. In each trial, the average values of cross-correlation and coherence function were calculated.

Cross-correlation is a time-domain method to characterize the correlated degree between two signals *x*(*t*) and *y*(*t*) by computing their correlation coefficients *ρ̂_xy_* at various time lags *k*:

(1) $${\hat{\rho }}_{xy}=\frac{{\hat{\sigma }}_{xy}(k)}{{\left[{\hat{\sigma }}_{x}(0){\hat{\sigma }}_{y}(0)\right]}^{1/2}},k=0,\pm 1,\pm 2,\cdots ,\pm K$$

The numerator is the cross-covariance function *σ̂_xy_*(*k*) defined as:

(2) $${\hat{\sigma }}_{xy}(k)=\{\begin{array}{ll} \frac{1}{n}\sum_{t=1}^{n-k}(x(t)-{\hat{\mu }}_{x})(y(t+k)-{\hat{\mu }}_{y}), & k\geq 0\\ \frac{1}{n}\sum_{t=1-k}^{n}(x(t)-{\hat{\mu }}_{x})(y(t+k)-{\hat{\mu }}_{y}), & k<0 \end{array}$$

where *μ̂_x_* an *μ̂_y_* are the mean values. The denominator is the square root of the products of auto-covariance functions. The *σ̂_x_*(*k*) is defined as:

(3) $${\hat{\sigma }}_{x}(k)=\frac{1}{n}\sum_{t=1}^{n-k}\left(x(t)-{\hat{\mu }}_{x})(x(t+k)-{\hat{\mu }}_{x}\right),\;\;\;k=0,1,2,\cdots K$$

The cross-correlation coefficient is bounded between −1 and +1. For two uncorrelated signals, the computed cross-correlogram is nearly flat and close to zero. For highly associated signals, their correlation coefficients can reach +1 or −1 for positive or negative associations, respectively [21]. In the present study, three pairs of activity-activity cross-correlations are calculated from full-wave rectified EMGs (RF-VM, RF-VL and VM-VL). The peak with maximum correlation around the zero lag and above upper 95% confidence interval, which has been defined as the significant level, were identified. The peak coefficient and absolute time lag of cross-correlation were calculated.

The activity-activity magnitude square coherence |*C_xy_* (*f*)|^2^ is the extension of cross-correlation and quantified in frequency domain. It is defined as:

(4) $$|C_{xy}(f){|}^{2}=\frac{|S_{xy}(f){|}^{2}}{S_{xx}(f)S_{yy}(f)}$$

where *S_xy_*(*f*) is the cross-spectrum between two signals x(t) and y(t), and *S_xx_*(*f*) and *S_yy_*(*f*) are the auto-spectra of x(t) and y(t), respectively. The coherence function provides a normative measurement of linear correlations between EMG signals over frequencies on a scale of 0 to 1, representing uncorrelated to highly associated conditions at the specified frequency.

The coherences derived from four step cycles. Each step cycle contained an ascent and a descent phase and each phase was about 1 s long. The data in the same phase, ascent or descent, of each of the step cycles were pooled together. The coherence spectra were computed from the three pairs of full-wave rectified EMGs (RF-VM, RF-VL and VM-VL) based on Welch’s periodogram using a 2048-point Hamming window with 75% overlapping, and the frequency resolution was 2.44 Hz. The coherence area in the alpha (8–12 Hz), beta (15–30 Hz) and gamma (30–60 Hz) bands were computed by summing the significant coherences (above upper 95% confidence interval of the coherence spectrum) and multiplied by frequency resolution. In order to assure that the inter-muscular coherence was not due to passive volume conduction, the coherence area in a high-gamma band (60–150 Hz) was also computed.

### 2.5. Statistical Analysis

One-way repeated measure ANOVA was used to compare the force of knee extensors and the rEMG of RF, VM, and VL between pre- and post-fatigue isometric MVCs. A paired t test was used to compare the coefficient and absolute time lag of cross-correlation, and coherence areas between the first and final trial of the stepping exercise. 95% confidence interval was used for cross-correlation and coherence function under the assumption that the muscle pair was independent. Statistical results were considered to be significant at *p* < *0.05*.

## 3. Results

**Figure 1.**
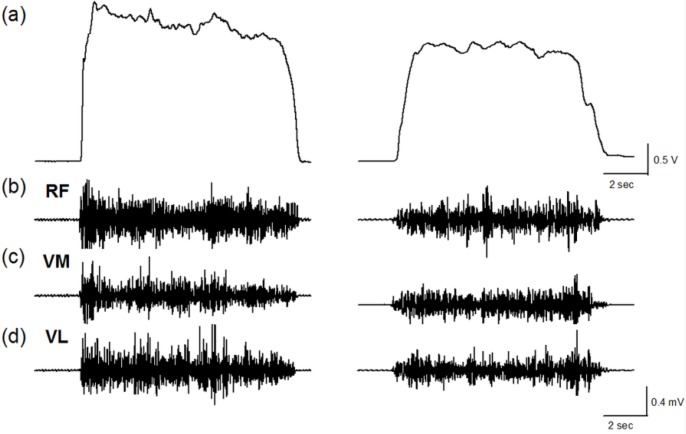
A single trial data of a representative subject during 10-sec isometric MVCs before (left column) and 1 min after (right column) the stepping exercise. (**a**) Force of knee extensors (**b**) electromyogram (EMG) of rectus femoris (RF) (**c**) EMG of vastus medialis (VM) (**c**) EMG vastus lateralis (VL).

**Figure 2.**
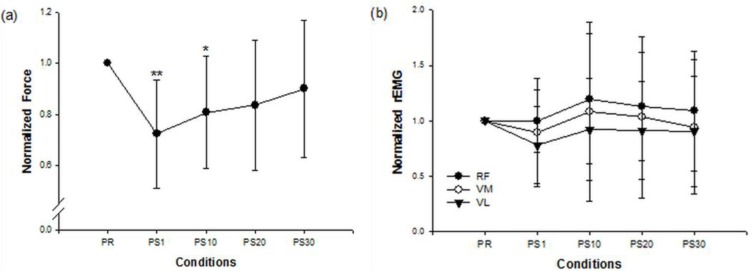
Force (**a**) and root-mean-square EMG (rEMG) (**b**) data were presented as relative values. Both have been normalized with respect to the reference values recorded during pre-fatigue isometric maximal voluntary contraction (MVC) (PR). The diagram showed the changes of the normalized force and rEMG during post-fatigue MVCs at 1 min (PS1), 10 min (PS10), 20 min (PS20) and 30 min (PS30) after stepping exercise. Normalized force and rEMG of VM and VL dropped shortly after stepping exercise and progressively recovered after 10 min. Asterisks showed the significant difference between conditions (* *p* < *0.05*; ** *p*< *0.01*).

Figure 1 shows the force and EMG data during an isometric MVC before and after the stepping exercise from a representative subject. Figure 2(a,b) display the changes of normalized force and rEMGs. The normalized force decreased to 72% at 1 min (*p* = *0.016*) and 81% at 10 min (*p* = *0.02*) after stepping exercise as compared with the pre-fatigue data. The result confirmed the muscle fatigue following the exhausting stepping exercise. A rapid recovery of the normalized force was observed at 20 min after stepping exercise. Although the normalized rEMG of RF showed a trend toward increasing 10 min after exercise, the change did not reach statistical significance (*p* = *0.291*). The normalized rEMG of VM and VL had no significantly change either.

**Figure 3.**
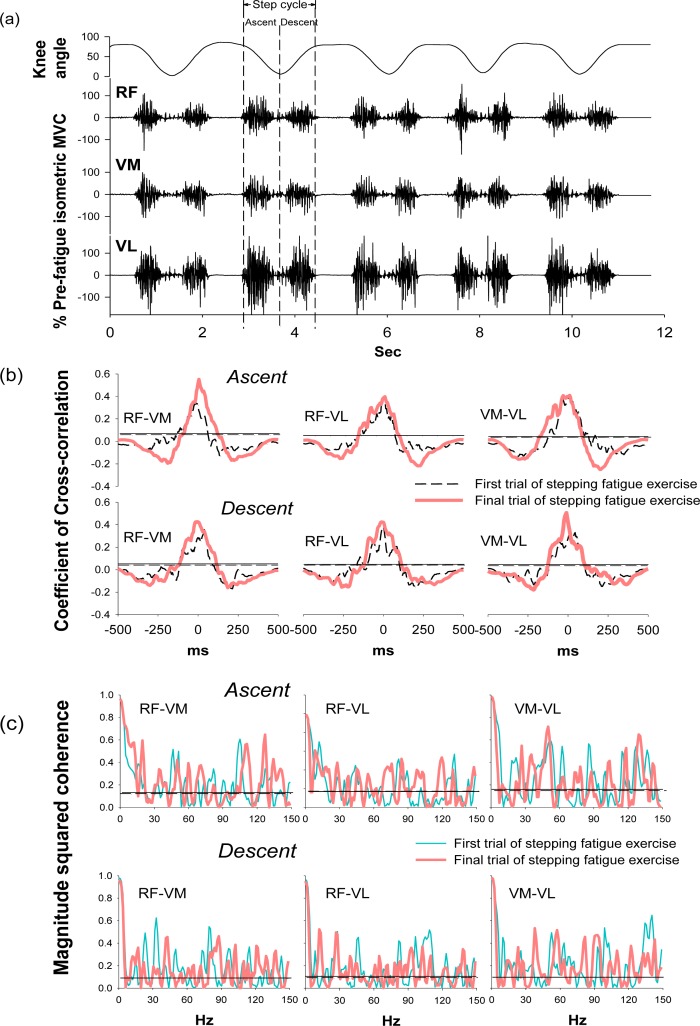
EMG signals from knee muscles of rectus femoris (RF), vastus medialis (VM) and vastus lateralis (VL) during stepping exercise and the corresponding changes in the range of motion of the knee joint from one representative subject (**a**). The diagram of cross-correlation (**b**) and coherence (**c**) for three muscle pairs of RF-VM, RF-VL and VM-VL during the first (bold dash line) and final trial (bold solid line) of stepping exercise. The horizontal lines in (b) and (c) are the averaged 95% confident intervals for the first (thin dash line) and final trial (thin solid line) of stepping exercise.

During the stepping exercise, the intermuscular cross-correlation and coherence changed along with the development of muscle fatigue as Figure 3 shows. The coefficients and the time lags of cross-correlation among three muscle pairs had no obvious change in the step ascent phase during fatigue (Table 2). However, in the step descent phase, the coefficient increased in both RF-VM and VM-VL muscle pairs.

The time lags decreased in all three muscle pairs from 38.10 ± 17.02 to 22.56 ± 18.89 for RF-VM (*p* = *0.044*), from 35.15 ± 22.24 to 26.29 ± 21.08 for RF-VL (*p* = *0.038*), and from 31.42 ± 24.08 to 17.31 ± 13.03 for VM-VL (*p* = *0.036*). These results suggested the significant change of intermuscular cross-correlation during the step descent phase after fatigue.

Coherence peaks were observed around 20–30 Hz and 38–50 Hz, which corresponded to the beta and gamma bands, respectively, in the first trial of the stepping exercise; however, diffused coherence bands presented in the end of the exercise, suggesting a change of intermuscular coherence after fatigue. In the step ascent phase, RF-VM showed significantly increased coherence areas in the beta band (from 3.38 ± 1.00 to 4.74 ± 1.97, *p* = *0.033*) and in the high-gamma band (14.63 ± 3.62 to 18.30 ± 3.68, *p* = *0.026*) after fatigue (Table 2). In the step descent phase, as muscles were fatigued, RF-VL had a significant increase in the coherence area in beta band (from 2.65 ± 1.05 to 4.46 ± 1.81, *p* = *0.017*). There was no significant change in the alpha band and gamma band (Table 2), suggesting the change of intermuscular coherence was specific to certain frequency bands. The spectral power in the same frequency bands was analyzed to evaluate if the change mirrors the changes observed in the same frequency bands of coherence. (Table 3) The result showed that beta band power did not increased as coherence and demonstrated non-mirror effect.

## 4. Discussion

As muscles were fatigued by the exhausting stepping exercise, both RF-VM and VM-VL showed increases of coefficients and the shortening of time lags. RF-VM and RF-VL also showed increases of coherence area in the beta band in the ascent and descent phases, respectively (Figure 3). These results indicated the fatigue-related increases of intermuscular cross-correlation and coherence in the knee synergistic muscles during dynamic and repetitive stretch-shortening contractions. When knee synergistic muscles have been in a fatigued state due to the repetitive contractions, the increases of intermuscular cross-correlation and coherence may be one of the compensatory strategies to decelerate neuromuscular failures and maintain muscle coordination for preventing injury [28]. Additionally, the beta-band intermuscular coherence are thought to be used for conveying cortical drives from the corticospinal tract. The boosting cortical drives in the beta band may associate with the enhancement of sensory-motor feedback during the generation of suppressed voluntary movements [14,29,30]. Therefore, in support of previous studies, the present results reflect that the increases of intermuscular cross-correlation and beta-band coherence may be a novel index for assessing the central control of motor function during the exhausting dynamic exercise.

The exhausting stepping exercise was confirmed to induce significant fatigue among subjects in this study. At the end of the stepping exercise, subjects showed significant declines in force and reported RPE scores of 20. RPE is common index of general fatigue level, and our previous work found that the increased RPE score may derive from the contributions of peripheral and central factors [5]. The peripheral factors contributing to fatigue include calcium ion accumulation [31], muscle injury, and neuromuscular transmission failure along the sarcolemma [32]. Alterations in the intracellular action potential, decreases in the motor-unit propagation velocity, and changes in motor-unit action potential shapes, phases duration and amplitudes [33–35] also contribute to the peripheral fatigue. One of the central factors is the recruitments of additional motor units. Our study found that the rEMG amplitude was not significantly decreased, suggesting the compensatory recruitments of additional motor units, which was also reported during sustained sub-maximal contractions [36], and if there was a drop out of motor units during fatigue, the amplitude of rEMG should be declined [37].

The increases of intermuscular cross-correlation of RF-VM and VM-VL are first, to our knowledge, found during the dynamic fatiguing exercise. Previous studies reported the increases of coefficients of intramuscular cross-correlation as muscles were fatigued by sustained sub-MVCs. The coefficient of intramuscular EMG of *biceps brachii* muscle increased from 0.23 to 0.25 with muscle fatigued by sustained 33% MVCs [38], and the intramuscular EMG of VL increased from 0.23 to 0.34 with muscles being fatigued by sustained 20% MVCs [24]. When the muscles were fatigued by sustained sub-MVCs, Dartnall *et al.* observed that the time lag of the intramuscular EMG of *biceps brachii* muscle decreased slightly from 18.8 ms to 18.4 ms [39]. The increase of coefficient and decrease of time lag related to the temporal and spatial summations in terms of muscle function as well as the increased common cortical drives, which further promotes the neural synchronization. With the impairment of corticospinal transmission, people with spinal cord injury and stroke were reported to have broad non-prominent central peaks without additional oscillatory signals among intramuscular EMG of *tibialis anterior* muscles [17,18]. Moreover, fatigue was also thought of as one of the factors disturbing the corticospinal transmission [40]. Therefore the increase of intermuscular cross-correlation in the present study might be a compensatory central control strategy for maintaining the coordination of knee synergistic muscles during fatigue.

In the frequency domain, the beta-band coherence of RF-VM in the ascent phase and RF-VL in the descent phase were significantly increased along with muscle fatigue. Previous studies have discovered an increase of intermuscular coherence in the beta band during sustained fatiguing sub-MVC [25], and the corticomuscular coherence analysis reveals that the transmission of oscillatory signals between cortex to muscles is beta frequency [14,19,41], the frequency that at ∼20 Hz is especially associated with voluntary movements. In patients with motor disability, the disappearance of beta-band coherence (15–30 Hz) were found in stroke and spinal cord injury [17,18], and the increase of beta-band coherence in patients with incomplete spinal cord injury following the 4-month treadmill training reflects the improvement in locomotor skills [42], suggesting that the beta-band oscillation associates with the recovery of corticospinal function. In support of these findings, because the fatigue-related disturbance of the corticospinal transmission was previously found, the increase of beta-band coherence of RF-VM and RF-VL in the present result further reflects the modulation of control of motor function during dynamic fatiguing exercise. The increases of beta-band coherence of RF-VM and RF-VL in the ascent and descent phase, respectively, suggested the distinct muscle synergy patterns during muscle concentric and eccentric contractions.

The present result shows no significant changes in alpha-band coherence along with the development of muscle fatigue. Although the previous study reported the increased alpha frequency oscillation during muscle fatigue [23], this may result from the non-physiological factors such as force fluctuations [24]. During the strenuous muscle contraction, the postural tremor of limb muscles may generate an oscillatory frequency around 10 Hz [43], but no significant changes of alpha-band coherence were seen in the present study, which might suggest that the postural tremor of knee muscles was not significantly associated with the development of fatigue during the stepping exercise. The result showed that beta band power did not increased as coherence and demonstrated non-mirror effect. Thus, passive volume conduction is least likely to the major influence factor. High-gamma band power was increased, especially in ascending phase. The high-gamma band reflects the waveform and conduction velocity of the action potential [44], which is different from the beta band which reflects the motor unit firing rate [45]. The changes in the high gamma power possibly reflected the phenomenon of recruiting additional motor units which was reported in fatiguing during submaximal contractions [46,47]. Whether the increase of high gamma band coherence is caused by physiology changes within muscle or by volume conduction is not clear.

In summary, the present study used a non-invasive method to find a potential index for muscle fatigue. The increased intermuscular cross-correlation and beta-band coherence in the knee synergistic muscles during dynamic and repetitive stretch-shortening contractions may associate with the development of muscle fatigue and help monitoring the coordination of synergistic muscles during dynamic fatiguing exercise. This method could also be applied with surface EMG sensors on monitoring training dosages in athletes or applied clinically on assessing patients who are prone to fatigue. The limitations of this study should be noticed. First, the intermuscular correlation and coherence were measured in muscles that control the same joint movements. Synergistic muscles that control different joints should be further studied. Second, because the present study does not measure the cortical activities, the results could not directly explain the fatigue-related increase of intermuscular cross correlation and beta-band coherence deriving from the alteration of corticomuscular signal transmission. The origins of the cortical areas which related to the increase of cross-correlation and beta-band coherence during dynamic fatiguing exercise are suggested to study in the future.

## Figures and Tables

**Table 1.** Age, sex, height and weight, and the numbers of stepping trials for stepping exercise for each subject.

**Subject**	**Age (y)**	**Sex**	**Height (cm)**	**Weight (kg)**	**Stepping Trials**
1	23	Male	176	78	25
2	22	Female	170	58	26
3	29	Male	163	57	27
4	29	Male	170	57	11
5	26	Female	163	55	16
6	25	Female	163	61	32
7	22	Female	158	44	20
8	22	Female	158	54	23
9	22	Male	168	66	18
10	20	Male	175	65	13
11	20	Male	175	60	13
Mean ± SD	23.6 ± 3.2	M:6/F:5	167.2 ± 6.6	59.6 ± 8.5	20.4 ± 6.8

**Table 2.** Comparison of the changes in the coefficient and time lag of cross-correlation, and coherence area in different frequency bands (alpha band: 8–12 Hz; beta band: 15–30 Hz; gamma band: 30–60 Hz; high-gamma band: 60–120 Hz) among three muscle pairs of RF-VM, RF-VL and VM-VL between the first and final trial of the exhausting stepping exercise. Asterisks showed the significant difference between the first and final trial (* *p* < *0.05*; ** *p* < *0.01*).

**Coefficient of Cross-Correlation**							**Time Lag of Cross-Correlation (ms)**						

	RF-VM		RF-VL		VM-VL			RF-VM		RF-VL		VM-VL	
	
	First trial	Final trial	First trial	Final trial	First trial	Final trial		First trial	Final trial	First trial	Final trial	First trial	Final trial
Ascent	0.45 ± 0.11	0.48 ± 0.09	0.45 ± 0.16	0.49 ± 0.08	0.48 ± 0.09	0.51 ± 0.08	Ascent	13.56 ± 8.86	11.00 ± 5.08	24.27 ± 20.83	15.92 ± 12.57	13.03 ± 7.25	12.75 ± 7.95
Descent	0.36 ± 0.10	0.43 ± 0.10 *	0.37 ± 0.10	0.39 ± 0.11	0.38 ± 0.08	0.46 ± 0.08 **	Descent	38.10 ± 17.02	22.56 ± 18.89 *	35.15 ± 22.24	26.29 ± 21.08 *	31.42 ± 24.08	17.31 ± 13.03 *

**Coherence Area**													

Bands	RF-VM		Ascent		VM-VL		Bands	RF-VM		Descent		VM-VL	
			RF-VL							RF-VL			
			
	First trial	Final trial	First trial	Final trial	First trial	Final trial		First trial	Final trial	First trial	Final trial	First trial	Final trial
Alpha	1.39 ± 0.64	1.32 ± 0.94	1.33 ± 0.89	1.36 ± 0.91	1.51 ± 0.76	1.24 ± 0.83	Alpha	0.93 ± 0.82	0.76 ± 0.46	0.95 ± 0.96	1.28 ± 0.96	0.89 ± 0.47	1.07 ± 0.92
Beta	3.38 ± 1.00	4.74 ± 1.97 *	4.65 ± 2.47	4.66 ± 1.65	4.41 ± 1.60	4.05 ± 1.90	Beta	4.29 ± 1.97	3.56 ± 1.56	2.65 ± 1.05	4.46 ± 1.81 *	4.25 ± 1.96	4.05 ± 2.12
Gamma	5.88 ± 1.79	5.55 ± 1.88	5.23 ± 2.60	5.34 ± 1.94	5.21 ± 1.97	5.51 ± 2.22	Gamma	5.87 ± 2.23	5.54 ± 1.96	5.07 ± 1.22	5.94 ± 2.84	5.55 ± 1.31	6.16 ± 2.24
High-gamma	14.63 ± 3.62	18.30 ± 3.68 *	17.76 ± 7.94	17.79 ± 5.02	14.97 ± 3.66	17.75 ± 2.80	High-gamma	15.08 ± 5.02	16.47 ± 3.72	17.15 ± 6.30	16.45 ± 4.36	16.89 ± 6.80	17.89 ± 5.42

**Table 3.** Comparison of the changes in the area under the power spectrum curve different frequency bands (alpha band: 8–12 Hz; beta band: 15–30 Hz; gamma band: 30–60 Hz; high-gamma band: 60–120 Hz) in three muscles RF, VM, and VL between the initial and final trial of the exhausting stepping exercise. Asterisks showed the significant difference between the first and final trial (* *p* < *0.05*; ** *p* < *0.01*).

**PSD Band Area**													
Bands	REC		Ascent		VL		Bands	REC		Descent		VL	
			VM							VM			
			
	First trial	Final trial	First trial	Final trial	First trial	Final trial		First trial	Final trial	First trial	Final trial	First trial	Final trial
Alpha	0.86 ± 0.86	0.90 ± 0.59	0.83 ± 0.48	0.70 ± 0.33	0.81 ± 0.68	0.71 ± 0.46	Alpha	1.15 ± 0.60	1.14 ± 0.88	1.14 ± 0.63	0.78 ± 0.43	1.16 ± 0.78	0.76 ± 0.39
Beta	5.50 ± 2.24	7.39 ± 2.71	7.18 ± 2.28	7.32 ± 2.68	6.80 ± 2.59	6.32 ± 3.00	Beta	8.11 ± 1.80	7.03 ± 1.33	8.03 ± 2.92	8.40 ± 2.32	7.83 ± 2.18	7.94 ± 2.21
Gamma	13.22 ± 1.91	15.18 ± 3.22	13.11 ± 2.58	13.56 ± 2.56	13.58 ± 2.56	14.00 ± 2.32	Gamma	12.75 ± 2.27	12.88 ± 2.89	14.16 ± 2.38	12.70 ± 2.31	12.96 ± 2.05	13.50 ± 3.15
High gamma	15.06 ± 4.20	22.21 ± 5.34 *	17.71 ± 5.43	22.95 ± 7.48 **	16.21 ± 5.20	21.74 ± 6.56 *	High gamma	18.76 ± 5.22	24.15 ± 4.96 *	22.21 ± 5.03	24.19 ± 7.10	19.46 ± 5.27	20.95 ± 7.07
